# Dynamic Ultrasound Assessment and Guided Medial Plantar Nerve Hydrodissection for Master Knot of Henry Syndrome

**DOI:** 10.3390/diagnostics14202266

**Published:** 2024-10-11

**Authors:** Wei-Ting Wu, Levent Özçakar, Ke-Vin Chang

**Affiliations:** 1 Department of Physical Medicine and Rehabilitation, National Taiwan University Hospital, Bei-Hu Branch, Taipei 100006, Taiwan; wwtaustin@yahoo.com.tw; 2 Department of Physical Medicine and Rehabilitation, College of Medicine, National Taiwan University, Taipei 100006, Taiwan; 3 Department of Physical and Rehabilitation Medicine, Hacettepe University Medical School, 06100 Ankara, Turkey; lozcakar@yahoo.com; 4 Center for Regional Anesthesia and Pain Medicine, Wang-Fang Hospital, Taipei Medical University, Taipei 110301, Taiwan

**Keywords:** ultrasonography, ankle, pain, entrapment, dextrose

## Abstract

A 27-year-old female presented with persistent right medial plantar pain that developed over six months following an ankle sprain. The pain, described as sharp and radiating to the toes, progressively worsened, affecting her ability to walk. An initial ultrasound examination suggested medial plantar nerve compression by a lipoma, prompting her referral for ultrasound-guided hydrodissection. During the pre-procedure assessment, sono-palpation (palpation using the ultrasound transducer) localized the pain to the Master Knot of Henry—where the medial plantar nerve, artery, and flexor tendons intersect. No lipoma but a normal fat pad was observed. Ultrasound-guided hydrodissection with 5% dextrose mixed with lidocaine and saline was performed. After two sessions, her pain significantly decreased, with her visual analogue scale score dropping from 8 to 5 after the first session and to 2 after the second, allowing her to resume normal activities. This case highlights the value of ultrasound in accurately diagnosing and treating conditions involving the Master Knot of Henry.

A 27-year-old female presented with right medial plantar pain persisting for six months. She reported that the pain had begun after an ankle sprain and had progressively worsened, eventually making walking difficult. The pain was described as sharp, radiating to the plantar surface of the toes. Ultrasound examination conducted at another hospital suggested compression of the medial plantar nerve by a lipoma, and she was referred for ultrasound-guided nerve hydrodissection.

Before the procedure, she experienced pain reproduction when the transducer was placed in the coronal plane over the dome of the medial foot arch. Dynamic flexion and extension of the big and second toes confirmed the pain originating at the Master Knot of Henry (Video S1), where the medial plantar artery, medial plantar nerve, flexor digitorum longus (FDL), and flexor hallucis longus (FHL) tendons intersect (Figure 1A,B). However, no lipoma but a normal fat pad was observed between the flexor digitorum brevis and abductor hallucis brevis muscles. Subsequently, ultrasound-guided hydrodissection using a mixture of 0.5 mL 50% dextrose, 2 mL 1% lidocaine, and 2.5 mL normal saline was performed using an in-plane lateral-to-medial approach (Figure 1C and Video S2). Five minutes post-injection, the injected foot appeared redder and warmer than the contralateral side. She was able to walk barefoot immediately after the procedure (Figure 1D). Following two sessions, her pain significantly reduced, with her visual analogue scale score decreasing from 8 to 5 after the first and to 2 after the second, enabling her to resume normal activities. No complications were observed after each injection.

The Master Knot of Henry [1] refers to a confined area in the plantar midfoot—located between the abductor hallucis muscle and the anatomical crossing of the FHL and FDL tendons (Figure 2). This restricted region is prone to compression, which can result in tendinopathy or even tears of the FHL and FDL tendons at the Knot of Henry. In some cases, tendinous connections between the FHL and FDL may be present [2]. The medial plantar nerve and the adjacent medial plantar artery are also at risk of compression in this area [3].

Ultrasound imaging is valuable for assessing conditions related to the Master Knot of Henry. Among symptomatic individuals, the most frequently observed cause is tenosynovitis of the FHL, characterized by thickening of the tendon sheath, fluid accumulation around the tendon, and increased blood flow to the affected area. Symptoms may also arise from a ganglion cyst (originating from the subtalar joint or a nearby tendon sheath), which typically appears as an anechoic structure with smooth and well-defined margins. Another potential cause is schwannoma of the medial plantar nerve, which may present as a fusiform or oval-shaped encapsulated mass connected to a nerve [4].

Dynamic ultrasound examination facilitated the identification of FDL and FHL tendons at the Master Knot of Henry [5]. To visualize the movement of the two tendons, we suggest to mobilize the second toe and then the first toe. If the first toe is mobilized first, the movement of the FHL may inadvertently cause the FDL to move as well, because the FHL lies directly beneath the FDL, leading the observer to mistakenly identify the FDL’s movement as that of the FHL. As no obvious pathologies were identified in either tendon in our case, irritation of the medial plantar nerve might be the best to explain the patient’s symptom.

Therefore, ultrasound-guided medial plantar nerve hydrodissection was performed. A 5% dextrose solution was used, as it is shown to be more effective than corticosteroids for hydro-dissecting the median nerve in carpal tunnel syndrome [6]. Since our injectate contained also lidocaine, the sympathetic tone of the medial plantar artery was reduced, leading to reactive vasodilation and subsequent erythematous changes in the skin. Of note, such a skin color change can also serve as an indicator of successful perineural injection. Furthermore, the decrease in pain may be mediated through selective mechanical denervation or neurotoxicity of the sensory component of the medial plantar nerve during hydrodissection, potentially due to the selective traumatic or neurotoxic effects of glucose/lidocaine on some sensory axons [7]. In conclusion, this case highlights the usefulness of ultrasound for both the assessment and guided injection of Master Knot of Henry syndrome.

## Figures and Tables

**Figure 1:**
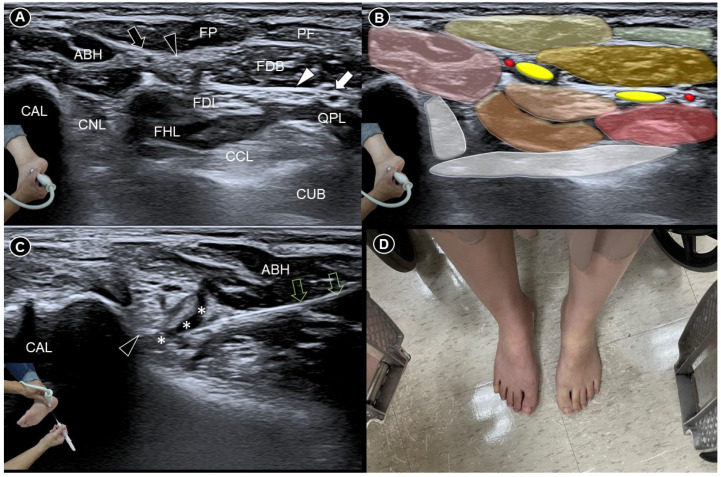
Ultrasound imaging (**A**) and schematic drawing (**B**) of the Master Knot of Henry. Ultrasound-guided medial plantar nerve hydrodissection (**C**), followed by erythematous skin changes in the injected foot (**D**). FHL, flexor hallucis longus tendon; FDL, flexor digitorum longus tendon; ABH, abductor hallucis; FDB, flexor digitorum brevis; QPL, quadratus plantae; CAL, calcaneus; CUB, cuboid; FP, fat pad; PF, plantar fascia; CCL, calcaneocuboid ligament; CNL, calcaneonavicular ligament; black arrowhead, medial plantar nerve; black arrow, medial plantar artery; white arrowhead, lateral plantar nerve; white arrow, lateral plantar artery; green void arrows, needle trajectory; asterisks, injectate.

**Figure 2:**
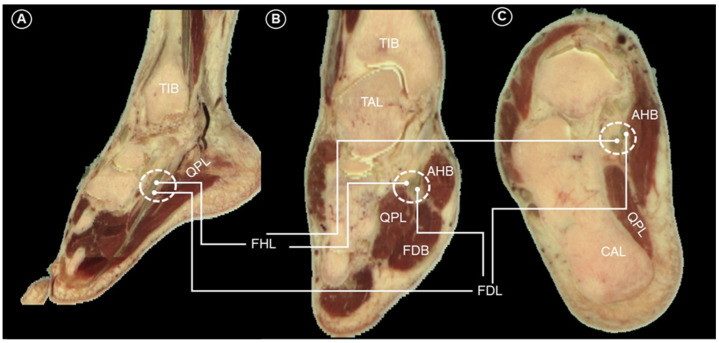
Sagittal (**A**), coronal (**B**), and horizontal (**C**) cadaveric sections illustrating the anatomy of the Master Knot of Henry (dashed circles). Images adapted from cadaveric images provided by the Visible Human Project^®^ of the National Library of Medicine. Excerpts featured in the VH Dissector are used with permission from Touch of Life Technologies Inc. FHL, flexor hallucis longus tendon; FDL, flexor digitorum longus tendon; AHB, abductor hallucis brevis; FDB, flexor digitorum brevis; QPL, quadratus plantae; CAL, calcaneus; TIB, tibia; TAL, talus.

## Data Availability

Data are contained within the main text of the manuscript.

## Supplementary Materials

The following supporting information can be downloaded at: https://www.mdpi.com/article/10.3390/diagnostics14202266/s1, Video S1: Ultrasound examination of the Master Knot of Henry; Video S2: Ultrasound guided injection on the Master Knot of Henry.
